# Sodium Chloride Increases Aβ Levels by Suppressing Aβ Clearance in Cultured Cells

**DOI:** 10.1371/journal.pone.0130432

**Published:** 2015-06-15

**Authors:** Xiao-Juan Cheng, Yuan Gao, Yu-Wu Zhao, Xiao-Dong Cheng

**Affiliations:** 1 Department of Neurology, Shanghai Jiao Tong University Affiliated Sixth People’s Hospital, Shanghai, China; 2 Department of Neurology & Institute of Neurology, Rui Jin Hospital affiliated to Shanghai Jiao Tong University School of Medicine, Shanghai, China; 3 Department of Neurology, First People’s Hospital of Yunnan Province affiliated to Kunming University of Science and Technology, Kunming, China; 4 School of Life Sciences and Technology, Tongji University. East Hospital Affiliated To Tongji University, Shanghai, China; Torrey Pines Institute for Molecular Studies, UNITED STATES

## Abstract

Recent studies suggest that high-salt diet is associated with cognitive decline in human and mouse. The fact that genetic factors account for less than 50% cases of sporadic Alzheimer’s disease (AD) highlights the important contribution of environmental factors, such as high-salt diet, in AD pathogenesis. However, whether and how high-salt diet fits the “amyloid cascade” hypothesis remains unexplored. Here, we show sodium chloride (NaCl) could increase Aβ levels in the medium of HEK293 cells overexpressing amyloid precursor protein (APP) or C99 fragment. NaCl treatment dose not affect APP level, gamma secretase level or activity. Instead, NaCl treatment suppresses the capacity of cells to clear Aβ and reduces Apolipoprotein E (ApoE) level. Finally, NaCl treated THP-1 or BV2 cells are inefficient in clearing Aβ when co-cultured with rat primary neurons. Our study suggests that high-salt diet may increase AD risk by directly modulating Aβ levels.

## Introduction

Alzheimer’s disease is a common neurodegenerative disease. Familial AD is caused by APP or presenilin (PS) mutations that lead to abnormal Aβ production. For sporadic AD which accounts for 90% of all AD cases, its underlining mechanism appears to be much more complex and only less than 50% of sporadic AD cases could be attributed to genetic factors [1]. It suggests environmental factors may play important roles in the pathogenesis of AD. Nevertheless, the dominant “amyloid cascade” hypothesis believes that Aβ plays a vital role in driving the pathogenesis of AD [2].

Several independent epidemiological investigations have shown that high-salt diet is associated with cognitive decline in human [3,4,5]. Recently, it’s found that high-salt diet could impair cognitive function in wild-type mice [6]. As cognitive decline is the core symptom of AD, these studies suggest that high-salt diet might be a risk factor for AD. In fact, high-salt diet would lead to hypertension, and then greatly increase the risk of vascular dementia (VaD) [7,8]. Interestingly, autopsy shows that many VaD patients also have Aβ plaques, the AD-like pathology, in their brains [9,10]. Therefore, the possible direct effects of high-salt diet on Aβ homeostasis and therefore the AD risk could not be underestimated and deserve further investigation.

Studies have shown high-salt diet has profound effects on peripheral blood immune cells in mice and these cells, although far away from the central nervous system (CNS), could play important roles in some nervous system diseases after they pass the blood-brain-barriers and infiltrate into CNS [11,12]. More specifically, macrophages in the peripheral blood could enter brain to affect Aβ level in AD mouse models [13,14,15]. The possible effects of high-salt diet on these immune cells and their contributions to AD were unclear. Based on the above studies, it’s possible that high-salt diet could affect the function of macrophages in mice and these macrophages could further modulate Aβ level in the brain. In the present study, we sought to investigate whether and how high-salt treatment could affect Aβ homeostasis in cell models of AD.

## Materials and Methods

### Cell cultures

HEK293 cell (human embryonic kidney cell), BV2 (murine microglial cell line) and THP-1 (human acute monocytic leukemia derived cell line) were from ATCC (American Type Culture Collection). HEK293-APP stable cell and HEK293-C99 stable cell were established as follows. HEK293 cells were transfected with pcDNA 3.1 vector overexpressing APP or C99. Stable clones were obtained after addition of G418 (final concentration is 500ug/ml) for 2 weeks. To maintain the stable cell lines, 500ug/ml of G418 was used. HEK293 cell, HEK293-APP stable cell, HEK293-C99 stable cell and BV2 (murine microglial cell line) were maintained in Dulbecco’s Modified Essential Medium (DMEM, GIBCO) with 10% Fetal bovine serum (FBS, GIBCO). THP-1 (human acute monocytic leukemia derived cell line) was maintained in RPMI1640 (GIBCO) with 10% FBS. Primary cortical neurons were dissected from embryonic day 17 (E17) brains of Sprague-Dawley rat or APP/PS1 mouse (#004462, Jackson laboratory) and cultured in Neurobasal (Invitrogen, 21103–049). All animal experiments were in accordance with the Institutional Animal Care and Use Committee of Shanghai Jiao Tong University, China. The study and protocols were approved by the Institutional Animal Care and Use Committee of Shanghai Jiao Tong University (Permit Number: SYXK 2011–0128). All efforts were made to minimize suffering of the animals.

### Co-culture system

The co-culture system consists of lower and upper chambers which are separated by a selectively permeable membrane with 0.4um-diameter pores (Corning, Transwell 3450). Rat primary neurons were plated in the lower chamber and maintained to 10 days in vitro (DIV10). Cell lines were plated in the upper chamber and were co-cultured with neurons after treated with normal or NaCl medium for indicated time.

### Reagents and antibodies

The following antibodies were used: APP from Invitrogen (13–0200, clone LN27), PS1 from Chemicon (MAB5232), nicastrin from Sigma (MAB5232), Pen2 from Invitrogen (36–7100) and ApoE from Calbiochem (178479). L685,458 (L1790) was from Sigma.

### Aβ ELISA

For cell lines, they were plated in 12-well plate and incubated with normal or NaCl medium (with additional 40mM NaCl) for 24h when they reach 80% confluence. For primary neurons from APP/PS1 mice, they were cultured in 12-well plate for 10 days in vitro (DIV 10) and incubated with normal or NaCl medium for 24h. To preclude that the effects of additional 40mM NaCl are unspecific results of increased ion concentration or osmotic pressure, additional 26.7mM MgCl2 (with equal ion concentration) or 80mM Mannitol (with equal osmotic pressure) were used as control solutions. After that, the medium was replaced by normal medium (600 ul for each well) for 12h before collection. The collected medium was centrifuged at 13200 rpm/min for 10 min to avoid cells and debris and stored at -80°C until ELISA experiments. Human Aβ42 and Aβ40 levels in the medium were quantified by ELISA kits (Invitrogen, KHB3482 and KHB3544). Rat Aβ derived from cultured neurons were quantified by ELISA kit (IBL, 27720). The Aβ levels in the culture medium are normalized to the total protein amount of the cells and then presented as fold changes relative to the control group.

### Western blot

Proteins were extracted from cell lines or cultured medium using sodium dodecyl sulfate lysis buffer (2% sodium dodecyl sulfate, 10% glycerol, 0.1 mM dithiothreitol, and 0.2 M Tris—HCl, pH 6.8). Protein samples were resolved by SDS—PAGE and analyzed by immunoblots.

### Gamma secretase activity assay

The human coding sequence of C99 was cloned into PET28 vector and expressed as fusion protein with His tag. C99-His recombinant protein was expressed by 1mM IPTG induction for 8 hours at 37°C in BL21 E. Coli. The recombinant protein was purified by Ni-column (QIAGEN) and serves as substrate. HEK293 cell line was homogenized and centrifuged at 13200 rpm/min for 15 min, and gamma secretases was reconstituted by resolving the pellet in 50 mM TrisHCL (pH 6.8), 2 mM EDTA and 0.25% CHAPSO (w/v). Twenty ug of protein was incubated with 2ug C99-His recombinant protein at 37°C for 4h. Then Aβ40 and Aβ42 levels were measured by ELISA.

### Aβ clearance assay

Cell lines were plated in 12-well plate and reached 80% confluence 24h later. Cells were treated with normal or NaCl medium for 24h. Then the culture medium was replaced by normal medium with 1ug/ml Aβ42. After 8h incubation, remaining Aβ42 in the medium was measured by ELISA.

### Annexin V-FITC Apoptosis assay

HEK293 cells were treated with normal or NaCl medium for indicated time and stained with Annexin V-FITC Apoptosis kit (abcam, ab14085) according to the manufacturer’s instruction. Then, the stained cells were analyzed by MoFlo XDP (Beckman Coulter, Inc).

### Statistical analysis

All data were presented as mean ± SEM. Statistical analysis was performed by two-tailed Student’s t test for two groups or One-way analysis of variance (ANOVA) followed by Newman-Keuls Multiple Comparison Test for more than two groups, using GraphPad Prism. Statistically significant differences were defined as P < 0.05. For all, *P<0.05, **P<0.01, ***P<0.001.

## Results

### NaCl increases Aβ levels in HEK293 cells and primary neurons from APP/PS1 mouse

**Fig 1:**
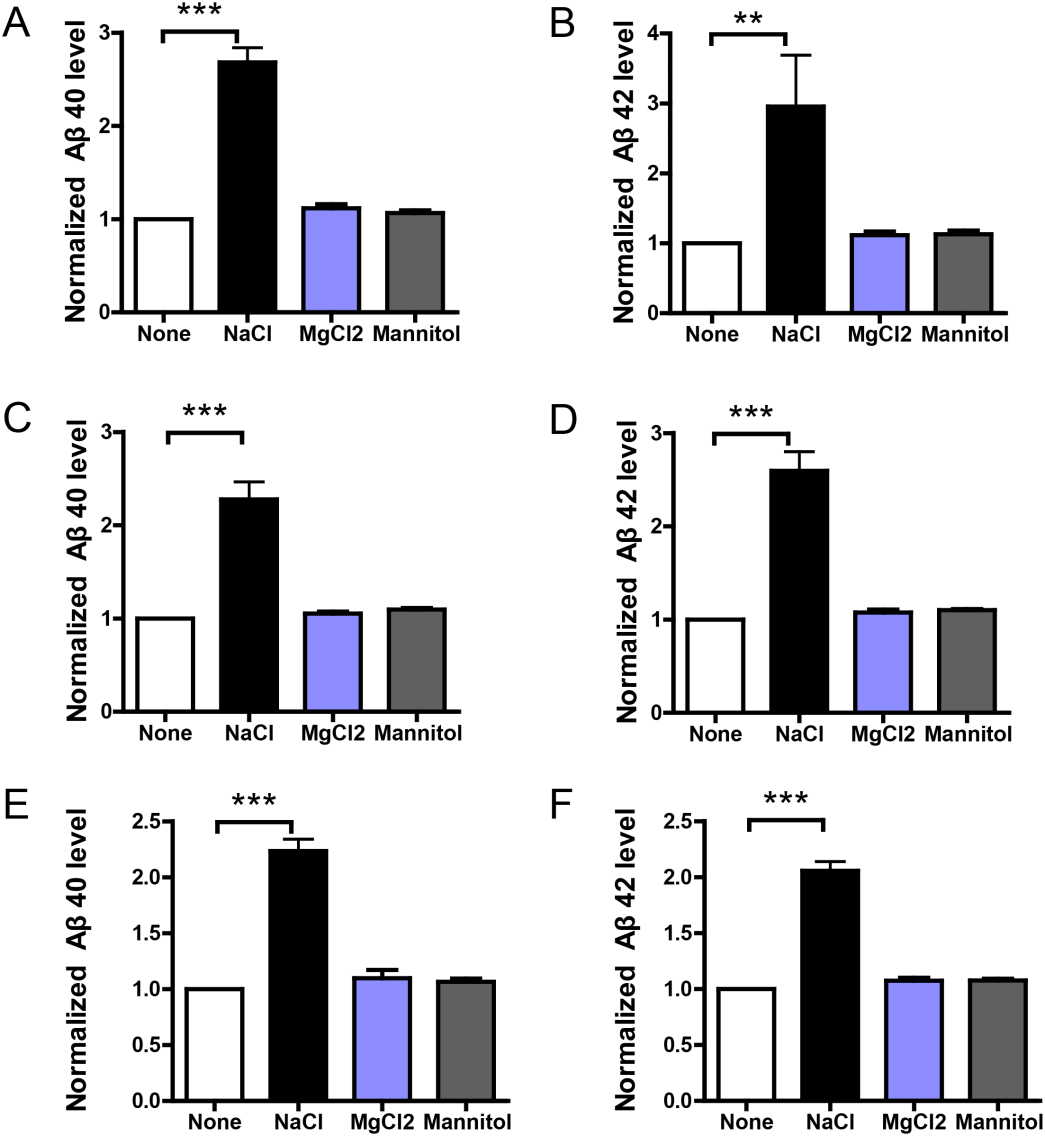
NaCl treatment increased Aβ levels in culture medium. HEK293 stable cells overexpressing APP (A and B) or C99 (C and D), or primary neurons from APP/PS1 mouse (E and F) were incubated with normal or NaCl medium for 24h and the medium were replaced by normal medium for another 12h. Then Aβ levels in the medium were measured by ELISA (n = 3). Data were presented as means ± s.e.m. of indicated numbers of independent experiments. **P<0.01, ***P<0.001.

To explore the possible effects of high-salt treatment, we define the condition of high-salt treatment as normal medium (DMEM for HEK293 and BV2, RPMI 1640 for THP-1, Neurobasal for primary neurons) with additional 40mM increase in NaCl concentration. HEK293 stable cells overexpressing APP or C99, and DIV10 primary neuron cultures from APP/PS1 mouse were incubated with normal or NaCl medium for 24h. To preclude that the effects of additional 40mM NaCl are unspecific results of increased ion concentration (the total ion concentration is increased by 80mM), we choose the normal medium with additional 26.7mM MgCl2 (the total ion concentration is also increased by 80mM) as a control solution for total ion concentration. Similarly, additional 40mM NaCl also increases osmotic pressure. To see if the effects of additional 40mM NaCl are simply resulted from increased osmotic pressure, we use normal medium with additional 80mM Mannitol (with the same osmotic pressure of 40mM NaCl) as a control solution for osmotic pressure. Then the medium was replaced by normal medium for 12h and Aβ levels in the medium were measured by ELISA. Results show that NaCl treatment greatly increased Aβ 40 and 42 levels in the medium (Fig 1). Importantly, the medium containing additional 26.7mM MgCl2 or 80mM mannitol could not affect Aβ levels in the medium. It indicates increased sodium, instead of increased chloride or osmolarity, is responsible for the effect of NaCl medium on Aβ levels. As Aβ levels are determined by the balance between Aβ production and clearance, the above results imply NaCl treatment might affect Aβ production or clearance. Further, given that C99 is the product of β-secretase cleavage of APP and serves as the direct substrate of γ secretase, our results suggest NaCl treatment might affect the γ-secretase cleavage of APP or Aβ clearance.

### NaCl dose not affect cell viability or γ secretase

**Fig 2:**
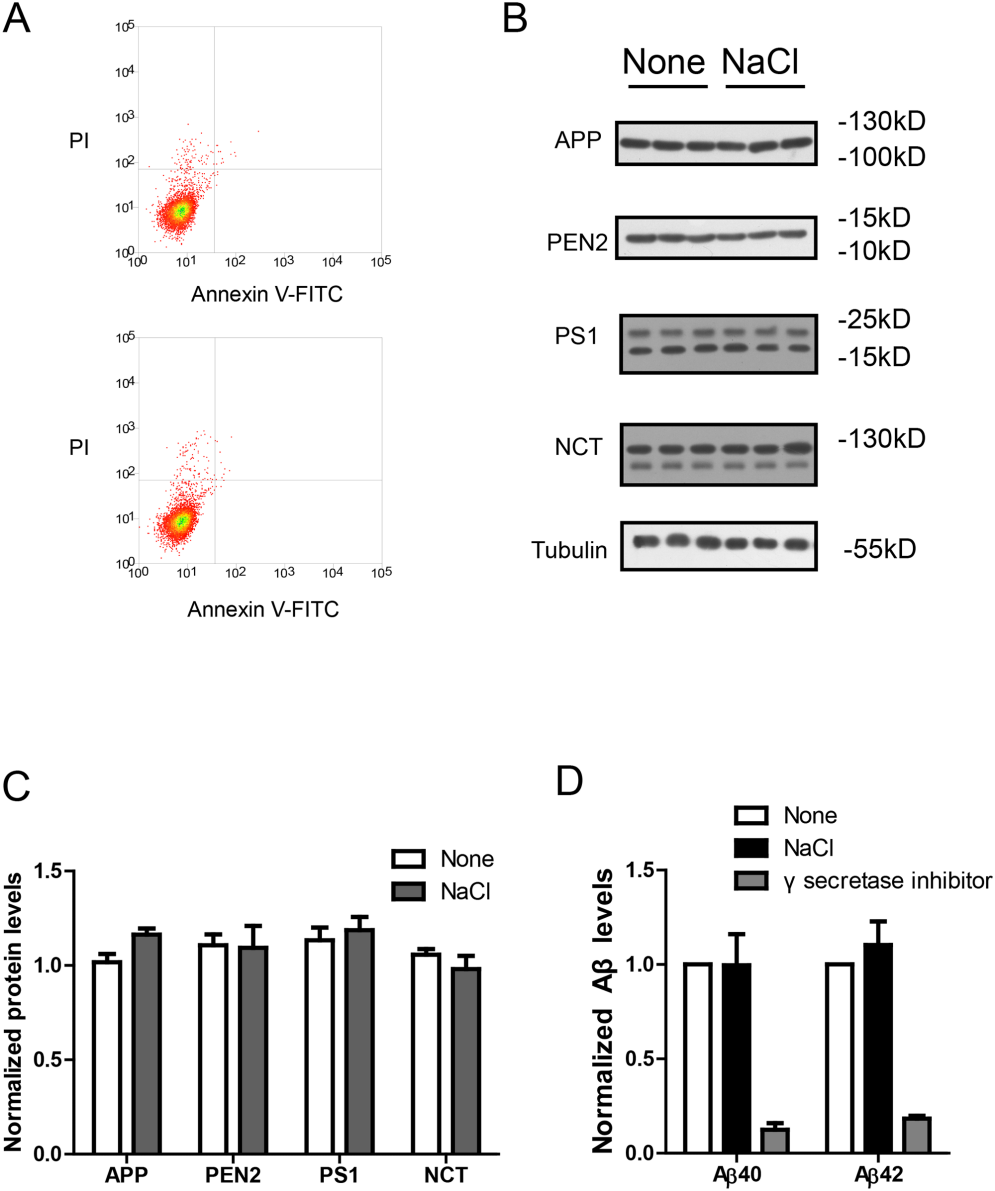
NaCl treatment did not affect gamma secretase. (A) FACS analysis showed the Annexin V-FITC/ PI staining of HEK293 cells treated with normal (up) or NaCl (low) medium. Western blots (B) and their quantification (C) showed the protein levels of APP and gamma secretase components (PS1, NCT, PEN2) in the lysate of HEK293 cells overexpressing APP after normal or NaCl treatment (n = 3). (D) Enzyme activity assay showed the gamma secretase activity of HEK293 cell lysate after treatment with normal, NaCl medium or gamma secretase inhibitor-L685,458 (n = 3). Data were presented as means ± s.e.m. of indicated numbers of independent experiments.

Next, we measured the possible cellular damage resulted from NaCl medium with the Annexin V-FITC apoptosis assay. The results show NaCl treatment for 24h in HEK293 cells did not result in cellular damage (Fig 2A). To explore the possible effects of NaCl treatment on γ-secretase cleavage of APP, we investigated whether the expression levels of APP or γ-secretase components were affected by NaCl treatment. Western blots (Fig 2B and 2C) show that NaCl treatment did not affect the protein levels of APP or γ-secretase components (PS1, NCT, PEN2). Moreover, we measured the enzyme activity of γ secretase under NaCl treatment in an in-vitro C99 assay [16]. The recombinant C99-His protein was used as the direct substrate of γ secretase and the membrane fractions from HEK293 cells treated with normal or NaCl medium were used as γ secretase. After incubation of the membrane fractions with C99-His protein, the Aβ levels were measured by ELISA and normalized to total protein amount. The normalized Aβ levels could reflect the γ-secretase activity. We found NaCl treatment did not affect γ-secretase activity (Fig 2D). In contrast, treatment with L685,458, a potent γ-secretase inhibitor, could abolish the γ-secretase activity. Theses results suggest NaCl treatment could increase Aβ levels without affecting cell viability, APP or γ secretase.

### NaCl suppresses Aβ clearance in HEK293, THP-1 and BV2 cells

**Fig 3:**
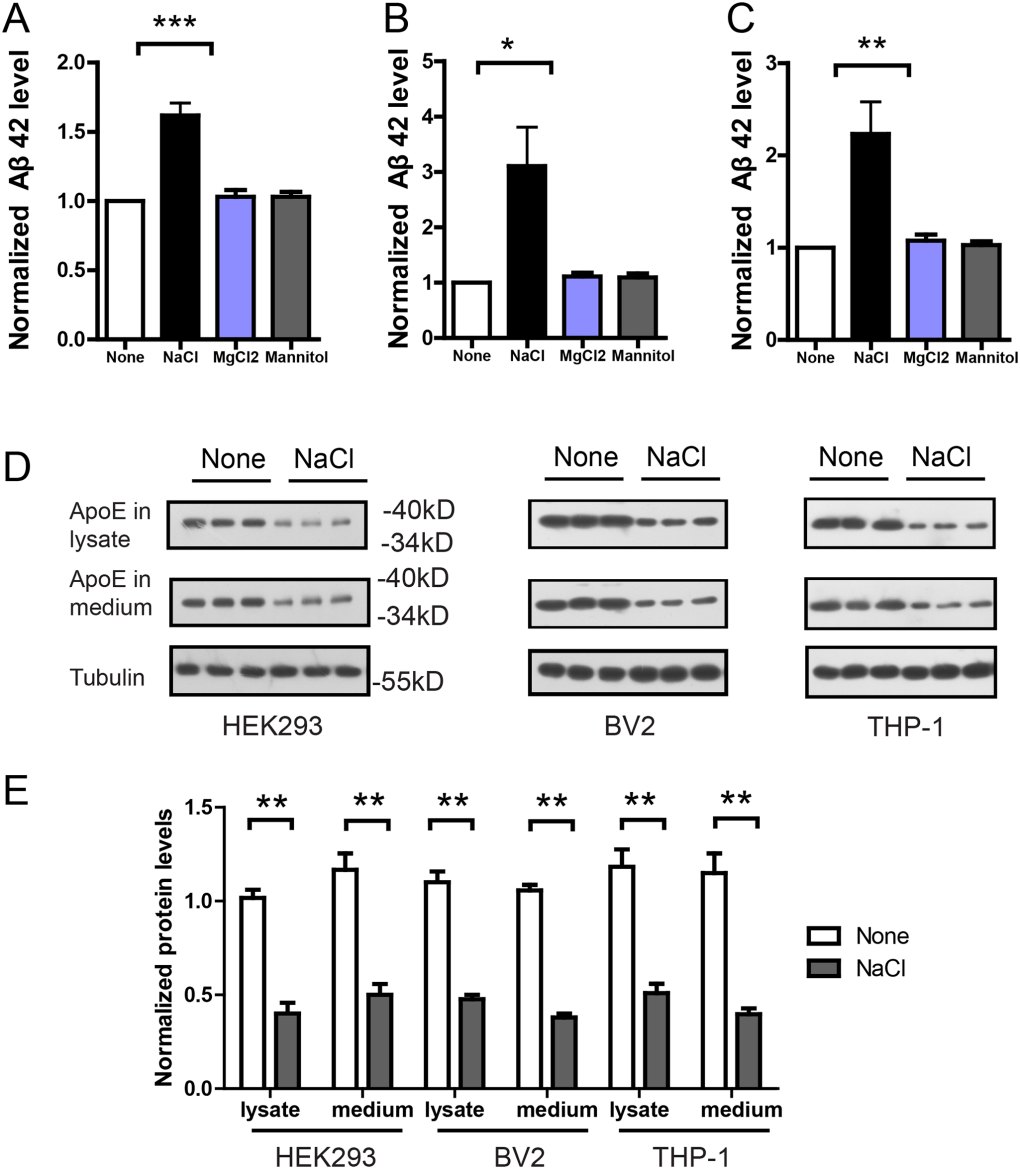
NaCl treatment suppressed Aβ clearance and reduced ApoE level. Aβ clearance assays were performed in HEK293 (A), BV2 (B) or THP-1 (C) cells. Normal or NaCl treated cells were incubated with 1ug/ml Aβ42 for 8h and the remaining Aβ42 in the medium was measured by ELISA (n = 3–5). Western blots (D) and their quantification (E) showed the protein level of ApoE in the lysates and medium of HEK293, BV2 or THP-1 cells after normal or NaCl treatment (n = 3). Data were presented as means ± s.e.m. of indicated numbers of independent experiments. *P<0.05, **P<0.01, ***P<0.001.

Then we reasoned that NaCl treatment could increase Aβ levels by suppressing its clearance. In the Aβ clearance assay [17], HEK293 cells were treated with normal or NaCl medium for 24h, and then the medium was replaced by normal medium containing 1ug/ml Aβ42. After 8h incubation, Aβ42 level in the medium was measured by ELISA. Indeed, Aβ42 level in the medium of NaCl treated cell was significantly higher than that of control cells (Fig 3A), indicating that NaCl treated cells was inefficient in Aβ clearance. In contrast, MgCl2 or Mannitol has no effects in the Aβ clearance assay.

In in-vivo condition, Aβ was cleared mainly by macrophages. Thus, we performed the same Aβ clearance assay in microglia cell line BV2 representing macrophages in the brain, and blood macrophage cell line THP-1. Similarly, Aβ42 levels in the medium of NaCl treated BV2 (Fig 3B) or THP-1 (Fig 3C) cells were significantly higher, indicating that NaCl treated BV2 or THP-1 cells were inefficient in Aβ clearance. We measured the viability of NaCl treated BV2 and THP-1 cells and found no significant effects of additional NaCl on cell viability with the Annexin V-FITC apoptosis assay (data not shown). In contrast, 26.7mM MgCl2 or 80mM Mannitol treated cells remained efficient in Aβ clearance. These results indicate increased sodium could suppress Aβ clearance. As ApoE plays an important role in Aβ clearance [17], we investigated whether ApoE level was affected by NaCl treatment. Indeed, in the lysates and medium of NaCl treated HEK293, BV2 or THP-1 cells, western blots show that ApoE level was significantly reduced (Fig 3D and 3E). Together, these results suggest that NaCl treatment could suppress Aβ clearance in HEK293, THP-1 and BV2 cells, possibly through down-regulating ApoE level.

### NaCl treated THP-1 or BV2 cells are inefficient in clearing Aβ derived from co-cultured neurons

**Fig 4:**
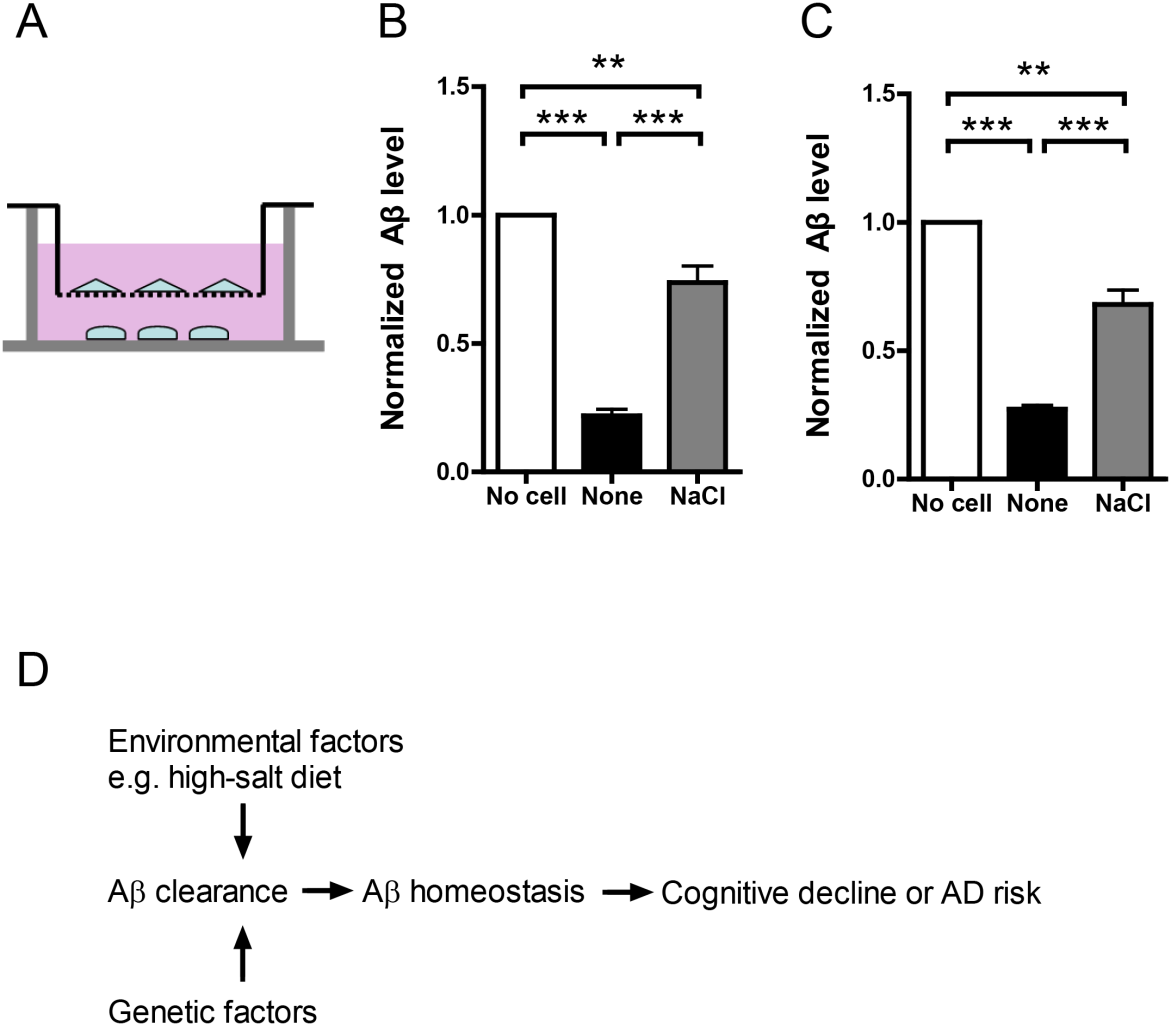
NaCl treatment suppressed Aβ clearance in co-culture system. (A) Rat primary neurons were plated in the lower chamber and treated cell lines were plated in the upper chamber. The lower and upper chambers were separated by a selectively permeable membrane with 0.4um-diameter pores. Normal or NaCl treated BV2 (B) or THP-1 (C) cells were co-cultured with neurons for 12h and the remaining Aβ in the lower chamber was measured by ELISA (n = 3). (D) Flow chart explains the potential involvement of high-salt diet in AD risk. Data were presented as means ± s.e.m. of indicated numbers of independent experiments. **P<0.01, ***P<0.001.

In in-vivo condition, neurons produce Aβ while macrophages clear Aβ. According to “Aβ cascade” hypothesis, dyshomeostasis of this tightly regulated balance would lead to AD. To recapitulate this complex process, we adopted an in-vitro co-culture system [18]. In this system, rat primary neurons (DIV10) were cultured in the lower chamber while normal or NaCl medium treated BV2 or THP-1 cells were cultured in the upper chamber (Fig 4A). There was a selectively permeable membrane with 0.4 um diameter between the lower and upper chambers, allowing free movement of substance smaller than 0.4um, such as Aβ. After 12h co-culture, Aβ level in the medium of the lower chamber was assayed by ELISA. As the results show, cultured rat neurons produced large amount of Aβ and its level was significantly reduced when co-cultured with normal medium treated BV2 or THP-1 cells. It indicates BV2 or THP-1 cells in the upper chamber could effectively clear Aβ in the lower chamber. However, when co-cultured with NaCl treated BV2 or THP-1 cells, Aβ level in the lower chamber was significantly higher (Fig 4B and 4C), indicating NaCl treated BV2 or THP-1 cells in the upper chamber were inefficient in Aβ clearance.

## Discussion

AD is an aging-related disease without effective therapy so far. The comprehensive understanding of its underlying mechanism is crucial for its prevention and treatment. The familial AD is caused by harmful mutations in APP or PS. For the sporadic AD, however, its mechanism of pathogenesis is much more complex. Genetic polymorphism in ApoE gene is the greatest risk factor in sporadic AD and ApoE4 genotype greatly increases AD risk [19]. But less than half of all sporadic AD patients were ApoE4 carriers. Thus, considerable efforts have been made to explore more potential genetic risk factors. Even through, the carriers of new identified risky single nucleotide polymorphism (SNP) are much scarce [1]. These studies suggest that nearly half of sporadic AD patients could not be attributed to genetic factors and potential environmental factors have to be taken into consideration.

Several studies have shown that high-salt diet, which is a common environmental factor in western countries as well as in more and more developing countries, is associated with cognitive decline [3,4,5]. Moreover, high-salt diet impairs cognitive function in mouse models [6]. These results point to a possibility that high-salt diet might increase AD risk. According to the dominant “amyloid cascade” hypothesis, AD is caused harmful Aβ accumulation. Thus we investigated whether and how could high-salt diet fit into the amyloid cascade. To mimic the effects of high-salt diet, we use DMEM or RPMI 1640 with additional 40mM NaCl to incubate cultured cells. Since the NaCl concentration in the normal DMEM or RPMI 1640 is about 140mM, the final NaCl concentration under our high-salt treatment is about 180mM. We choose this concentration because in certain tissues harbouring immune cells such as macrophages, NaCl concentration could be higher than 180mM in high-salt diet fed mice [20,21]. Also, recent studies suggest that additional 40mM NaCl could produce profound effects on cultured peripheral blood immune cells [11,12]. Thus, we choose this in-vitro condition of additional 40mM NaCl to mimic the in-vivo condition of high-salt diet fed mice. Our results show that NaCl treatment could increase Aβ levels in cells overexpressing APP or C99, indicating Aβ production or clearance was affected. APP is cleaved by beta secretase into C99 and then C99 is cleaved by gamma secretase to release Aβ. The results that substrate level (APP) and gamma secretase level or activity remained unchanged imply that Aβ clearance, but not Aβ production, was affected by high-salt treatment. Indeed, in the Aβ clearance assay, NaCl treated HEK293, BV2 or THP-1 cells became inefficient in Aβ clearance. Finally, to test the effects of NaCl treatment in a more physiological and in-vivo condition, we adopted a co-culture system consisting of macrophages in the upper chamber and primary neurons in the lower chamber. Similarly, NaCl treated BV2 or THP-1 cells were inefficient in Aβ clearance.

Our study provides a link between high-salt diet and Aβ in cultured cells. It may contribute to the association of high-salt diet with cognitive decline in human. As Aβ plays a key role in driving the pathogenesis of AD and brain Aβ levels are associated with cognitive decline in AD and normal aging, we speculate that high-salt diet might modulate Aβ levels to affect the cognitive decline and therefore AD risk (Fig 4D). It widens our understanding of AD pathogenesis and highlights the importance of healthy life style in AD prevention or reducing AD risk. But there are some limitations in our study. First, the direct association of high-salt diet with AD risk has not been established by epidemiological investigation. Second, the effects of high-salt treatment were investigated in cultured cells which might be quite different from the in-vivo condition. In conclusion, we report that high-salt treatment increases Aβ levels by suppressing Aβ clearance in cultured cells. To understand the mechanism underlying the association between the high-salt diet and the cognitive decline, it requires further in-vivo investigation in animal models in the future.
